# Supplementary material for: Scaling neural machine translation to 200 languages
Source: Nature. 2024 Jun 5;630(8018):841–6. doi: 10.1038/s41586-024-07335-x (PMC11208141; doi:10.1038/s41586-024-07335-x)
Supplement: Supplementary file 1 — This file contains Supplementary Information Sections A–K and Supplementary References – see Supplementary Contents page for details. [file 41586_2024_7335_MOESM1_ESM.pdf]

nature portfolio

https://doi.org/10.1038/s41586-024-07335-x

# Supplementary information

# Scaling neural machine translation to 200 languages

In the format provided by the authors and unedited

# Supplementary information

- We summarize information about each of our 200 supported languages in Appendix A.
- We discuss more details on the creation of NLLB-Seed in Appendix B, including source sentence selection and translation workflow.
- Additional details on the FLORES-200 human evaluation work can be found in Appendix C.
- Appendix D provides statistics about our 200-language training dataset, and shares additional ablation experiments on the effect of using different data sources on performance.
- We detail in Appendix E our dense model architecture and its MoE equivalent. We also enclose our curriculum learning buckets for the final NLLB-200 model.
- Appendix F provides additional procedural and annotator details for our human evaluation work (XSTS).
- We discuss the technical limitations of this work in Appendix G.
- Finally, model and data cards can be found in Appendices H to K

# A Languages

| Code | Language | Script | Family | Subgrouping | 🌐 | Res. | Specification |
|---|---|---|---|---|---|---|---|
| ace_Arab[NEW] | **Acehnese** | Arabic | Austronesian | Malayo-Polynesian | ✗ | Low | North Acehnese |
| ace_Latn[NEW] | **Acehnese** | Latin | Austronesian | Malayo-Polynesian | ✗ | Low | North Acehnese |
| acm_Arab[NEW] | **Mesopotamian Arabic** | Arabic | Afro-Asiatic | Semitic | ✗ | Low | Baghdadi |
| acq_Arab[NEW] | **Ta'izzi-Adeni Arabic** | Arabic | Afro-Asiatic | Semitic | ✗ | Low | |
| aeb_Arab[NEW] | **Tunisian Arabic** | Arabic | Afro-Asiatic | Semitic | ✗ | Low | Derja |
| afr_Latn | **Afrikaans** | Latin | Indo-European | Germanic | 🌐 | High | |
| ajp_Arab[NEW] | **South Levantine Arabic** | Arabic | Afro-Asiatic | Semitic | ✗ | Low | Ammani |
| aka_Latn[NEW] | **Akan** | Latin | Atlantic-Congo | Kwa Volta-Congo | ✗ | Low | Asante |
| amh_Ethi | **Amharic** | Ge'ez | Afro-Asiatic | Semitic | 🌐 | Low | Addis Ababa |
| apc_Arab[NEW] | **North Levantine Arabic** | Arabic | Afro-Asiatic | Semitic | ✗ | Low | |
| arb_Arab | **Modern Standard Arabic** | Arabic | Afro-Asiatic | Semitic | 🌐 | High | |
| arb_Latn[NEW] | **Modern Standard Arabic** | Latin | Afro-Asiatic | Semitic | ✗ | Low | |
| ars_Arab[NEW] | **Najdi Arabic** | Arabic | Afro-Asiatic | Semitic | ✗ | Low | |
| ary_Arab[NEW] | **Moroccan Arabic** | Arabic | Afro-Asiatic | Semitic | ✗ | Low | |
| arz_Arab[NEW] | **Egyptian Arabic** | Arabic | Afro-Asiatic | Semitic | ✗ | Low | |
| asm_Beng | **Assamese** | Bengali | Indo-European | Indo-Aryan | 🌐 | Low | Eastern |
| ast_Latn | **Asturian** | Latin | Indo-European | Italic | ✗ | Low | Central |
| awa_Deva[NEW] | **Awadhi** | Devanagari | Indo-European | Indo-Aryan | ✗ | Low | Ayodhya |
| ayr_Latn[NEW] | **Central Aymara** | Latin | Aymaran | Central Southern Aymara | 🌐 | Low | Aymara La Paz jilata |
| azb_Arab[NEW] | **South Azerbaijani** | Arabic | Turkic | Common Turkic | ✗ | Low | Tabrizi |
| azj_Latn | **North Azerbaijani** | Latin | Turkic | Common Turkic | 🌐 | Low | Shirvan |
| bak_Cyrl[NEW] | **Bashkir** | Cyrillic | Turkic | Common Turkic | 🌐 | Low | Literary |
| bam_Latn[NEW] | **Bambara** | Latin | Mande | Western Mande | 🌐 | Low | |
| ban_Latn[NEW] | **Balinese** | Latin | Austronesian | Malayo-Polynesian | ✗ | Low | |
| bel_Cyrl | **Belarusian** | Cyrillic | Indo-European | Balto-Slavic | 🌐 | Low | Central |
| bem_Latn[NEW] | **Bemba** | Latin | Atlantic-Congo | Benue-Congo | ✗ | Low | Central |
| ben_Beng | **Bengali** | Bengali | Indo-European | Indo-Aryan | 🌐 | High | Rarhi |
| bho_Deva[NEW] | **Bhojpuri** | Devanagari | Indo-European | Indo-Aryan | 🌐 | Low | |
| bjn_Arab[NEW] | **Banjar** | Arabic | Austronesian | Malayo-Polynesian | ✗ | Low | Banjar Kuala |
| bjn_Latn[NEW] | **Banjar** | Latin | Austronesian | Malayo-Polynesian | ✗ | Low | Banjar Kuala |
| bod_Tibt[NEW] | **Standard Tibetan** | Tibetan | Sino-Tibetan | Bodic | 🌐 | Low | Lhasa |
| bos_Latn | **Bosnian** | Latin | Indo-European | Balto-Slavic | 🌐 | High | |
| bug_Latn[NEW] | **Buginese** | Latin | Austronesian | Malayo-Polynesian | ✗ | Low | Bone |
| bul_Cyrl | **Bulgarian** | Cyrillic | Indo-European | Balto-Slavic | 🌐 | High | |
| cat_Latn | **Catalan** | Latin | Indo-European | Italic | 🌐 | High | |
| ceb_Latn | **Cebuano** | Latin | Austronesian | Malayo-Polynesian | 🌐 | Low | |
| ces_Latn | **Czech** | Latin | Indo-European | Balto-Slavic | 🌐 | High | |
| cjk_Latn[NEW] | **Chokwe** | Latin | Atlantic-Congo | Benue-Congo | ✗ | Low | |
| ckb_Arab | **Central Kurdish** | Arabic | Indo-European | Iranian | 🌐 | Low | |
| crh_Latn[NEW] | **Crimean Tatar** | Latin | Turkic | Common Turkic | ✗ | Low | |
| cym_Latn | **Welsh** | Latin | Indo-European | Celtic | 🌐 | Low | Y Wyndodeg |
| dan_Latn | **Danish** | Latin | Indo-European | Germanic | 🌐 | High | |
| deu_Latn | **German** | Latin | Indo-European | Germanic | 🌐 | High | |
| dik_Latn[NEW] | **Southwestern Dinka** | Latin | Nilotic | Western Nilotic | ✗ | Low | Rek |
| dyu_Latn[NEW] | **Dyula** | Latin | Mande | Western Mande | ✗ | Low | |
| dzo_Tibt[NEW] | **Dzongkha** | Tibetan | Sino-Tibetan | Bodic | ✗ | Low | |
| ell_Grek | **Greek** | Greek | Indo-European | Graeco-Phrygian | 🌐 | High | |
| eng_Latn | **English** | Latin | Indo-European | Germanic | 🌐 | High | |
| epo_Latn[NEW] | **Esperanto** | Latin | Constructed | Esperantic | 🌐 | Low | |
| est_Latn | **Estonian** | Latin | Uralic | Finnic | 🌐 | High | |
| eus_Latn[NEW] | **Basque** | Latin | Basque | – | 🌐 | High | |
| ewe_Latn[NEW] | **Ewe** | Latin | Atlantic-Congo | Kwa Volta-Congo | 🌐 | Low | Aŋlo |
| fao_Latn[NEW] | **Faroese** | Latin | Indo-European | Germanic | 🌐 | Low | |
| fij_Latn[NEW] | **Fijian** | Latin | Austronesian | Malayo-Polynesian | 🌐 | Low | Bau |
| fin_Latn | **Finnish** | Latin | Uralic | Finnic | 🌐 | High | |
| fon_Latn[NEW] | **Fon** | Latin | Atlantic-Congo | Kwa Volta-Congo | ✗ | Low | |
| fra_Latn | **French** | Latin | Indo-European | Italic | 🌐 | High | |
| fur_Latn[NEW] | **Friulian** | Latin | Indo-European | Italic | ✗ | Low | Central |
| fuv_Latn | **Nigerian Fulfulde** | Latin | Atlantic-Congo | North-Central Atlantic | ✗ | Low | Sokoto |
| gla_Latn[NEW] | **Scottish Gaelic** | Latin | Indo-European | Celtic | ✗ | Low | Northern Hebrides |
| gle_Latn | **Irish** | Latin | Indo-European | Celtic | 🌐 | Low | |
| glg_Latn | **Galician** | Latin | Indo-European | Italic | 🌐 | Low | |
| grn_Latn[NEW] | **Guarani** | Latin | Tupian | Maweti-Guarani | 🌐 | Low | |
| guj_Gujr | **Gujarati** | Gujarati | Indo-European | Indo-Aryan | 🌐 | Low | Amdavadi/Surti |
| hat_Latn[NEW] | **Haitian Creole** | Latin | Indo-European | Italic | 🌐 | Low | |
| hau_Latn | **Hausa** | Latin | Afro-Asiatic | Chadic | 🌐 | Low | |
| heb_Hebr | **Hebrew** | Hebrew | Afro-Asiatic | Semitic | 🌐 | High | |
| hin_Deva | **Hindi** | Devanagari | Indo-European | Indo-Aryan | 🌐 | High | |
| hne_Deva[NEW] | **Chhattisgarhi** | Devanagari | Indo-European | Indo-Aryan | ✗ | Low | |
| hrv_Latn | **Croatian** | Latin | Indo-European | Balto-Slavic | 🌐 | High | |
| hun_Latn | **Hungarian** | Latin | Uralic | – | 🌐 | High | |
| hye_Armn | **Armenian** | Armenian | Indo-European | Armenic | 🌐 | Low | Yerevan |
| ibo_Latn | **Igbo** | Latin | Atlantic-Congo | Benue-Congo | 🌐 | Low | Central |
| ilo_Latn[NEW] | **Ilocano** | Latin | Austronesian | Malayo-Polynesian | 🌐 | Low | |
| ind_Latn | **Indonesian** | Latin | Austronesian | Malayo-Polynesian | 🌐 | High | |
| isl_Latn | **Icelandic** | Latin | Indo-European | Germanic | 🌐 | High | |
| ita_Latn | **Italian** | Latin | Indo-European | Italic | 🌐 | High | |
| jav_Latn | **Javanese** | Latin | Austronesian | Malayo-Polynesian | 🌐 | Low | |
| jpn_Jpan | **Japanese** | Japanese | Japonic | Japanesic | 🌐 | High | |
| kab_Latn[NEW] | **Kabyle** | Latin | Afro-Asiatic | Berber | ✗ | Low | North Eastern |
| kac_Latn[NEW] | **Jingpho** | Latin | Sino-Tibetan | Brahmaputran | ✗ | Low | |
| kam_Latn | **Kamba** | Latin | Atlantic-Congo | Benue-Congo | ✗ | Low | Machakos |

| Code | Language | Script | Family | Subgrouping | 🌐 | Res. | Specification |
|---|---|---|---|---|---|---|---|
| kan_Knda | **Kannada** | Kannada | Dravidian | South Dravidian | 🌐 | Low | Central |
| kas_Arab[NEW] | **Kashmiri** | Arabic | Indo-European | Indo-Aryan | ✗ | Low | Kishtwari |
| kas_Deva[NEW] | **Kashmiri** | Devanagari | Indo-European | Indo-Aryan | ✗ | Low | Kishtwari |
| kat_Geor | **Georgian** | Georgian | Kartvelian | Georgian-Zan | 🌐 | Low | Kartlian |
| knc_Arab[NEW] | **Central Kanuri** | Arabic | Saharan | Western Saharan | ✗ | Low | Yerwa |
| knc_Latn[NEW] | **Central Kanuri** | Latin | Saharan | Western Saharan | ✗ | Low | Yerwa |
| kaz_Cyrl | **Kazakh** | Cyrillic | Turkic | Common Turkic | 🌐 | High | |
| kbp_Latn[NEW] | **Kabiyè** | Latin | Atlantic-Congo | North Volta-Congo | ✗ | Low | Kwɛ |
| kea_Latn[NEW] | **Kabuverdianu** | Latin | Indo-European | Italic | ✗ | Low | Sotavento |
| khm_Khmr | **Khmer** | Khmer | Austroasiatic | Khmeric | 🌐 | Low | Central |
| kik_Latn[NEW] | **Kikuyu** | Latin | Atlantic-Congo | Benue-Congo | ✗ | Low | Southern |
| kin_Latn[NEW] | **Kinyarwanda** | Latin | Atlantic-Congo | Benue-Congo | 🌐 | Low | |
| kir_Cyrl | **Kyrgyz** | Cyrillic | Turkic | Common Turkic | 🌐 | Low | Northern |
| kmb_Latn[NEW] | **Kimbundu** | Latin | Atlantic-Congo | Benue-Congo | ✗ | Low | |
| kmr_Latn[NEW] | **Northern Kurdish** | Latin | Indo-European | Iranian | 🌐 | Low | |
| kon_Latn[NEW] | **Kikongo** | Latin | Atlantic-Congo | Benue-Congo | ✗ | Low | |
| kor_Hang | **Korean** | Hangul | Koreanic | Korean | 🌐 | High | |
| lao_Laoo | **Lao** | Lao | Tai-Kadai | Kam-Tai | 🌐 | Low | Vientiane |
| lij_Latn[NEW] | **Ligurian** | Latin | Indo-European | Italic | ✗ | Low | Zeneise |
| lim_Latn[NEW] | **Limburgish** | Latin | Indo-European | Germanic | ✗ | Low | Maastrichtian |
| lin_Latn | **Lingala** | Latin | Atlantic-Congo | Benue-Congo | 🌐 | Low | |
| lit_Latn | **Lithuanian** | Latin | Indo-European | Balto-Slavic | 🌐 | High | |
| lmo_Latn[NEW] | **Lombard** | Latin | Indo-European | Italic | ✗ | Low | Western |
| ltg_Latn[NEW] | **Latgalian** | Latin | Indo-European | Balto-Slavic | ✗ | Low | Central |
| ltz_Latn | **Luxembourgish** | Latin | Indo-European | Germanic | 🌐 | Low | |
| lua_Latn[NEW] | **Luba-Kasai** | Latin | Atlantic-Congo | Benue-Congo | ✗ | Low | |
| lug_Latn | **Ganda** | Latin | Atlantic-Congo | Benue-Congo | 🌐 | Low | |
| luo_Latn | **Luo** | Latin | Nilotic | Western Nilotic | ✗ | Low | |
| lus_Latn[NEW] | **Mizo** | Latin | Sino-Tibetan | Kuki-Chin-Naga | 🌐 | Low | Aizawl |
| lvs_Latn | **Standard Latvian** | Latin | Indo-European | Balto-Slavic | 🌐 | High | |
| mag_Deva[NEW] | **Magahi** | Devanagari | Indo-European | Indo-Aryan | ✗ | Low | Gaya |
| mai_Deva[NEW] | **Maithili** | Devanagari | Indo-European | Indo-Aryan | 🌐 | Low | |
| mal_Mlym | **Malayalam** | Malayalam | Dravidian | South Dravidian | 🌐 | Low | |
| mar_Deva | **Marathi** | Devanagari | Indo-European | Indo-Aryan | 🌐 | Low | Varhadi |
| min_Arab[NEW] | **Minangkabau** | Arabic | Austronesian | Malayo-Polynesian | ✗ | Low | Agam-Tanah Datar |
| min_Latn[NEW] | **Minangkabau** | Latin | Austronesian | Malayo-Polynesian | ✗ | Low | Agam-Tanah Datar |
| mkd_Cyrl | **Macedonian** | Cyrillic | Indo-European | Balto-Slavic | 🌐 | High | |
| plt_Latn[NEW] | **Plateau Malagasy** | Latin | Austronesian | Malayo-Polynesian | 🌐 | Low | Merina |
| mlt_Latn | **Maltese** | Latin | Afro-Asiatic | Semitic | 🌐 | High | |
| mni_Beng[NEW] | **Meitei** | Bengali | Sino-Tibetan | Kuki-Chin-Naga | ✗ | Low | |
| khk_Cyrl | **Halh Mongolian** | Cyrillic | Mongolic-Khitan | Mongolic | 🌐 | Low | |
| mos_Latn[NEW] | **Mossi** | Latin | Atlantic-Congo | North Volta-Congo | ✗ | Low | Ouagadougou |
| mri_Latn | **Maori** | Latin | Austronesian | Malayo-Polynesian | 🌐 | Low | Waikato-Ngapuhi |
| mya_Mymr | **Burmese** | Myanmar | Sino-Tibetan | Burmo-Qiangic | 🌐 | Low | Mandalay-Yangon |
| nld_Latn | **Dutch** | Latin | Indo-European | Germanic | 🌐 | High | |
| nno_Latn[NEW] | **Norwegian Nynorsk** | Latin | Indo-European | Germanic | ✗ | Low | |
| nob_Latn | **Norwegian Bokmål** | Latin | Indo-European | Germanic | 🌐 | Low | |
| npi_Deva | **Nepali** | Devanagari | Indo-European | Indo-Aryan | 🌐 | Low | Eastern |
| nso_Latn | **Northern Sotho** | Latin | Atlantic-Congo | Benue-Congo | 🌐 | Low | |
| nus_Latn[NEW] | **Nuer** | Latin | Nilotic | Western Nilotic | ✗ | Low | |
| nya_Latn | **Nyanja** | Latin | Atlantic-Congo | Benue-Congo | 🌐 | Low | |
| oci_Latn | **Occitan** | Latin | Indo-European | Italic | ✗ | Low | |
| gaz_Latn[NEW] | **West Central Oromo** | Latin | Afro-Asiatic | Cushitic | 🌐 | Low | |
| ory_Orya | **Odia** | Oriya | Indo-European | Indo-Aryan | 🌐 | Low | Baleswari (Northern) |
| pag_Latn[NEW] | **Pangasinan** | Latin | Austronesian | Malayo-Polynesian | ✗ | Low | |
| pan_Guru | **Eastern Panjabi** | Gurmukhi | Indo-European | Indo-Aryan | 🌐 | Low | Majhi |
| pap_Latn[NEW] | **Papiamento** | Latin | Indo-European | Italic | ✗ | Low | Römer-Maduro-Jonis |
| pes_Arab | **Western Persian** | Arabic | Indo-European | Iranian | 🌐 | High | |
| pol_Latn | **Polish** | Latin | Indo-European | Balto-Slavic | 🌐 | High | |
| por_Latn | **Portuguese** | Latin | Indo-European | Italic | 🌐 | High | Brazil |
| prs_Arab[NEW] | **Dari** | Arabic | Indo-European | Iranian | 🌐 | Low | Kabuli |
| pbt_Arab | **Southern Pashto** | Arabic | Indo-European | Iranian | 🌐 | Low | Literary |
| quy_Latn[NEW] | **Ayacucho Quechua** | Latin | Quechuan | Chinchay | 🌐 | Low | Southern Quechua |
| ron_Latn | **Romanian** | Latin | Indo-European | Italic | 🌐 | High | |
| run_Latn[NEW] | **Rundi** | Latin | Atlantic-Congo | Benue-Congo | ✗ | Low | |
| rus_Cyrl | **Russian** | Cyrillic | Indo-European | Balto-Slavic | 🌐 | High | |
| sag_Latn[NEW] | **Sango** | Latin | Atlantic-Congo | North Volta-Congo | ✗ | Low | |
| san_Deva[NEW] | **Sanskrit** | Devanagari | Indo-European | Indo-Aryan | 🌐 | Low | |
| sat_Olck[NEW] | **Santali** | Ol Chiki | Austroasiatic | Mundaic | ✗ | Low | |
| scn_Latn[NEW] | **Sicilian** | Latin | Indo-European | Italic | ✗ | Low | Literary Sicilian |
| shn_Mymr[NEW] | **Shan** | Myanmar | Tai-Kadai | Kam-Tai | ✗ | Low | |
| sin_Sinh[NEW] | **Sinhala** | Sinhala | Indo-European | Indo-Aryan | 🌐 | Low | |
| slk_Latn | **Slovak** | Latin | Indo-European | Balto-Slavic | 🌐 | High | |
| slv_Latn[NEW] | **Slovenian** | Latin | Indo-European | Balto-Slavic | 🌐 | High | |
| smo_Latn[NEW] | **Samoan** | Latin | Austronesian | Malayo-Polynesian | 🌐 | Low | |
| sna_Latn | **Shona** | Latin | Atlantic-Congo | Benue-Congo | 🌐 | Low | |
| snd_Arab | **Sindhi** | Arabic | Indo-European | Indo-Aryan | 🌐 | Low | Vicholi |
| som_Latn | **Somali** | Latin | Afro-Asiatic | Cushitic | 🌐 | Low | Nsom |
| sot_Latn[NEW] | **Southern Sotho** | Latin | Atlantic-Congo | Benue-Congo | 🌐 | High | |
| spa_Latn | **Spanish** | Latin | Indo-European | Italic | 🌐 | High | Latin American |
| als_Latn[NEW] | **Tosk Albanian** | Latin | Indo-European | Albanian | 🌐 | High | |
| srd_Latn[NEW] | **Sardinian** | Latin | Indo-European | Italic | ✗ | Low | Logudorese and Campidanese |
| srp_Cyrl | **Serbian** | Cyrillic | Indo-European | Balto-Slavic | 🌐 | Low | |

| Code | Language | Script | Family | Subgrouping | 🌐 | Res. | Specification |
|---|---|---|---|---|---|---|---|
| ssw_Latn$^{\text{NEW}}$ | **Swati** | Latin | Atlantic-Congo | Benue-Congo | ✗ | Low | |
| sun_Latn$^{\text{NEW}}$ | **Sundanese** | Latin | Austronesian | Malayo-Polynesian | 🌐 | Low | |
| swe_Latn | **Swedish** | Latin | Indo-European | Germanic | 🌐 | High | |
| swh_Latn | **Swahili** | Latin | Atlantic-Congo | Benue-Congo | 🌐 | High | Kiunguja |
| szl_Latn$^{\text{NEW}}$ | **Silesian** | Latin | Indo-European | Balto-Slavic | ✗ | Low | |
| tam_Taml | **Tamil** | Tamil | Dravidian | South Dravidian | 🌐 | Low | Chennai |
| tat_Cyrl$^{\text{NEW}}$ | **Tatar** | Cyrillic | Turkic | Common Turkic | 🌐 | Low | Central and Middle |
| tel_Telu | **Telugu** | Telugu | Dravidian | South Dravidian | 🌐 | Low | Coastal |
| tgk_Cyrl | **Tajik** | Cyrillic | Indo-European | Iranian | 🌐 | Low | |
| tgl_Latn | **Tagalog** | Latin | Austronesian | Malayo-Polynesian | 🌐 | High | |
| tha_Thai | **Thai** | Thai | Tai-Kadai | Kam-Tai | 🌐 | High | |
| tir_Ethi$^{\text{NEW}}$ | **Tigrinya** | Geez | Afro-Asiatic | Semitic | 🌐 | Low | |
| taq_Latn$^{\text{NEW}}$ | **Tamasheq** | Latin | Afro-Asiatic | Berber | ✗ | Low | Kal Ansar |
| taq_Tfng$^{\text{NEW}}$ | **Tamasheq** | Tifinagh | Afro-Asiatic | Berber | ✗ | Low | Kal Ansar |
| tpi_Latn$^{\text{NEW}}$ | **Tok Pisin** | Latin | Indo-European | Germanic | ✗ | Low | |
| tsn_Latn$^{\text{NEW}}$ | **Tswana** | Latin | Atlantic-Congo | Benue-Congo | ✗ | High | Sehurutshe |
| tso_Latn$^{\text{NEW}}$ | **Tsonga** | Latin | Atlantic-Congo | Benue-Congo | 🌐 | Low | |
| tuk_Latn$^{\text{NEW}}$ | **Turkmen** | Latin | Turkic | Common Turkic | 🌐 | Low | Teke |
| tum_Latn$^{\text{NEW}}$ | **Tumbuka** | Latin | Atlantic-Congo | Benue-Congo | ✗ | Low | Rumphi |
| tur_Latn | **Turkish** | Latin | Turkic | Common Turkic | 🌐 | High | |
| twi_Latn$^{\text{NEW}}$ | **Twi** | Latin | Atlantic-Congo | Kwa Volta-Congo | 🌐 | Low | Akuapem |
| tzm_Tfng$^{\text{NEW}}$ | **Central Atlas Tamazight** | Tifinagh | Afro-Asiatic | Berber | ✗ | Low | |
| uig_Arab$^{\text{NEW}}$ | **Uyghur** | Arabic | Turkic | Common Turkic | 🌐 | Low | |
| ukr_Cyrl | **Ukrainian** | Cyrillic | Indo-European | Balto-Slavic | 🌐 | High | |
| umb_Latn | **Umbundu** | Latin | Atlantic-Congo | Benue-Congo | ✗ | Low | |
| urd_Arab | **Urdu** | Arabic | Indo-European | Indo-Aryan | 🌐 | Low | Lashkari |
| uzn_Latn | **Northern Uzbek** | Latin | Turkic | Common Turkic | 🌐 | High | |
| vec_Latn$^{\text{NEW}}$ | **Venetian** | Latin | Indo-European | Italic | ✗ | Low | Venice |
| vie_Latn | **Vietnamese** | Latin | Austroasiatic | Vietic | 🌐 | High | |
| war_Latn$^{\text{NEW}}$ | **Waray** | Latin | Austronesian | Malayo-Polynesian | ✗ | Low | Tacloban |
| wol_Latn | **Wolof** | Latin | Atlantic-Congo | North-Central Atlantic | ✗ | Low | Dakkar |
| xho_Latn | **Xhosa** | Latin | Atlantic-Congo | Benue-Congo | 🌐 | High | Ngqika |
| ydd_Hebr$^{\text{NEW}}$ | **Eastern Yiddish** | Hebrew | Indo-European | Germanic | 🌐 | Low | Hasidic |
| yor_Latn | **Yoruba** | Latin | Atlantic-Congo | Benue-Congo | 🌐 | Low | Oyo and Ibadan |
| yue_Hant$^{\text{NEW}}$ | **Yue Chinese** | Han (Traditional) | Sino-Tibetan | Sinitic | 🌐 | Low | |
| zho_Hans | **Chinese** | Han (Simplified) | Sino-Tibetan | Sinitic | 🌐 | High | |
| zho_Hant | **Chinese** | Han (Traditional) | Sino-Tibetan | Sinitic | 🌐 | High | |
| zsm_Latn | **Standard Malay** | Latin | Austronesian | Malayo-Polynesian | 🌐 | High | |
| zul_Latn | **Zulu** | Latin | Atlantic-Congo | Benue-Congo | 🌐 | High | |

**Table 4**: No Language Left Behind languages: We display the language *Code*, language name, *Script*, and language *Family*. The symbol 🌐 indicates machine translation support by Google and/or Microsoft (as of July 2022), whereas ✗ indicates support by neither. *Res.* indicates if we classify the language as high or low-resource. *Specification* contains, if available, additional information on the language variant collected in Flores-200. The superscript$^{\text{NEW}}$ indicates new languages added to Flores-200 compared to Flores-101.

# B NLLB-Seed dataset

Machine learning is notoriously data-hungry, leading to many research areas aimed at reducing the amount of required supervision. Recent advances in zero-shot learning [5, 64, 73, 74] and self-supervised learning [75–77], for instance, seek to reduce this reliance. However, generation tasks like translation are unlikely to reach the desired quality levels without some starter data. For instance, producing a good translation without seeing a minimum number of sentences in a new language is challenging. Similarly, it may be difficult to classify which language a sentence is in without seeing reliable examples of text in different languages. To this end, we create NLLB-Seed, a set of professionally translated sentences in the Wikipedia domain. NLLB-Seed comprises around six thousand sentences in 39 languages.

Such a data set has numerous potential uses. For instance, NLLB-Seed's target-side data in various languages can be deployed for language identification model building. The data set can also be used for its aligned bitext to train, for example, translation models. Another option is to use NLLB-Seed for domain finetuning, such as adapting general-purpose translation models to the Wikipedia domain.

### *Source sentence selection*

Data for NLLB-Seed was sampled from Wikimedia's *List of articles every Wikipedia should have*,[17] a collection of 10,000 Wikidata IDs corresponding to notable topics in different fields of knowledge and human activity. These are split into 11 categories such as *People*, *History*, *Philosophy and Religion*, and *Geography*. We uniformly sampled a subset of IDs from which we would draw data and mapped these to the corresponding English Wikipedia articles. From each of these articles, we sampled data that would be sent to translators. Instead of extracting individual sentences, which would have left translators with little context to work with, we chose to sample triplets of contiguous sentences, ensuring no more than one triplet per article was used (similar to Flores-200).

Like Flores-200, NLLB-Seed's source data is English-centric and sampled from English Wikipedia.[18] This has an important effect—the content reflects what Wikipedia editors find is relevant for English Wikipedia and likely does not cover a diverse spread of content from different cultures. Furthermore, the target text in NLLB-Seed is ultimately translated by humans and thus potentially contains effects of translationese (often defined as awkward, unnatural, or overly literal translations) [78].

### *Translation workflow*

Script, specification, spelling, and translation approaches were first established against Flores-200. Translators referenced these linguistic alignments while working on seed data translations. The data sets were translated directly from English for 39 languages while two Arabic script languages (Acehnese and Banjar) and Tamasheq in Tifinagh script were transliterated from their respective Latin script data sets (first

[17] https://meta.wikimedia.org/wiki/List_of_articles_every_Wikipedia_should_have/Expanded
[18] Note: There is no overlap between the sentences in Flores-200 and NLLB-Seed.

translated from English).[19] Following translation or transliteration was a linguistic quality assessment phase in which the completed data sets were checked against the linguistic alignments from FLORES-200, along with automatic quality control checks.

We note that NLLB-Seed has a key distinction compared to evaluation benchmarks such as Flores-200. Critically, NLLB-Seed is meant to be used for *training* rather than *model evaluation*. Due to this difference, NLLB-Seed does not go through the human quality assurance process present in Flores-200.

# C Human evaluation details

The final human quality test encompassed a 20% assessment by independent reviewers from a language service provider (LSP). The reviewers assessed translation errors at the sentence level, and the translation quality score per language was determined based on the number of errors identified by the reviewers. The following errors were examined: grammar, punctuation, spelling, capitalization, addition or omission of information, mistranslation, unnatural translation, untranslated text, and register. Each error was also associated with a severity level—minor, major, and critical. The overall score is constructed by tallying these different error types. The acceptable translation quality score was set at 90%. It is also important to note that there was first an initial alignment between the translators and LSP on the approach to take for each language. In cases of large disagreements, translators were also allowed to arbitrate with the reviewers to further align their understanding of translation quality. This was especially helpful for languages with lower levels of standardization.

# D Data technical details

To train NLLB-200, we leveraged three different types of bitexts:

### *Primary bitexts*

We use a set of publicly available parallel corpora from a variety of sources, including NLLB-Seed (Appendix I). We added a total of 661 sets of primary bitext data. We chose all English-centric sets when available and also added non-English-centric pairs if they had a low resource language as source, target, or both. Table 5 provides further information on the list of public bitext corpora we used for training.

### *Mined bitexts*

We used bitext corpora retrieved by large-scale *bitext mining*, as detailed in Section 2.1.2. We added mined data for a total of 784 directions. These included all English-centric directions and a subset of non-English-centric directions. Non-English-centric mined data effectively improves the performance of multilingual translation systems [1]. However, having 200 languages implies approximately 40,000 non-English-centric pairs, and adding all the pairs could be detrimental (as some pairs do not have

[19] We had a specific process for Ligurian: half the data for Ligurian were first translated from English to Italian, then translated from Italian to Ligurian, while the other half was translated directly from English. As we were lucky to have a native Ligurian speaker, we developed this process to improve quality.

high-quality mined bitexts). To select based on projected quality, we first picked directions with a `xsim` error rate of under 5. As a further restriction, we added mining data primarily for pairs containing low-resource languages within a given language family or a geographical region. This is an imperfect approximation to ensure improved transfer learning between similar languages.

***Back-translated bitexts***

Back-translated data provides a form of weak supervision, which is crucial for improving the translation performance of low-resource languages. Combining back-translation data generated from multiple sources improves the performance of a translation model due to increased back-translation diversity. Following this, we generated back-translated data from two models: **(1)** a multilingual neural machine translation model (MmtBT) and **(2)** a set of bilingual statistical machine translation models (SmtBT). We used monolingual data for a total of 192 languages to generate back-translated bitexts.

We share below the full list of bitexts used for training.

- Primary: https://github.com/facebookresearch/fairseq/tree/nllb/examples/nllb/modeling/scripts/flores200/lang_pairs_primary.txt
- Mined: https://github.com/facebookresearch/fairseq/tree/nllb/examples/nllb/modeling/scripts/flores200/lang_pairs_mine.txt
- Primary+Mined: https://github.com/facebookresearch/fairseq/tree/nllb/examples/nllb/modeling/scripts/flores200/lang_pairs_primary_mine.txt
- Primary+Mined+MmtBT+SmtBT: https://github.com/facebookresearch/fairseq/tree/nllb/examples/nllb/modeling/scripts/flores200/lang_pairs.txt

## D.1 Effect of using different data sources on performance

We expected to see cumulative benefits by combining different sources of data. We empirically explore this hypothesis in this section.

***Experimental Setup***

We trained dense 3.3B Transformer encoder-decoder models with model dimension 2048, FFN dimension 8192, 16 attention heads, and 48 layers (24 encoder, 24 decoder) for these data ablation experiments. We trained these models on three sets of data: **(1)** Primary, **(2)** Primary+Mined, and **(3)** Primary+Mined+MmtBT+SmtBT to compare the cumulative improvements coming from adding each source of data. All models were trained for a total of 300k iterations, and we report the results with best chrF++ score checkpoints.

***Results***

In Figure 6, we show the impact of adding different data sources over Primary data. We aggregated results over language pair type and resource level. We observe that across all language pairs, performance improves significantly by adding Mined data and further by adding MmtBT+SmtBT back-translated data. Focusing our observation on resource levels, we observe that low-resource languages improve more than

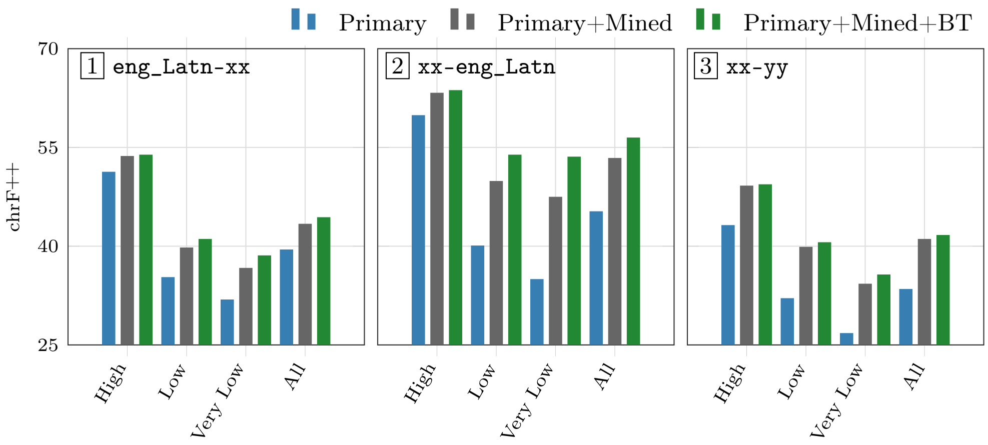


**Fig. 6**: Comparing model performance when trained on data from various sources. We observe significant improvements in adding mined and back-translated data for all types of language pairs and resource levels.

high-resource languages. This is not surprising, as high-resource languages already have significant amounts of Primary bitext data publicly available.

***Impact of mining and back-translation on very low-resource languages***

Looking deeper at the results, we investigated how mined and back-translated data sources impact very low-resource languages. We define *very low-resource* as languages with fewer than 100K unique sentence pairs across all language pairings available in public bitext corpora, with 84 total. On aggregate, our proposed techniques of mining and back-translation improved low-resource and very low-resource language directions significantly (see Figure 6). Most prominently, very low-resource into English directions improved by +12.5 chrF++ with mined data and +6.1 chrF++ with additional back-translation data, with an overall improvement of +18.6 chrF++.

Similarly, we observe that out-of-English directions improve by +4.7 chrF++ when adding mined data and +1.9 chrF++ when adding back-translated data, with an overall improvement of +6.6 chrF++. For non-English-centric pairs, we see an improvement of +7.5 chrF++ when adding mined data and +1.4 chrF++ when adding back-translated data, with an overall improvement of +8.9 chrF++. These results show that our improvements in bitext mining and back-translation increase the data quantity and quality for low-resource languages often underserved or excluded by existing translation systems.

## D.2 The 200 language dataset

Combining multiple sources of data, our final data set covers 200 languages.[20] The data set comprises primary bitext for 661 language pairs, mined bitext for 784 language

[20]Two languages in Flores-200, `arb_Latn` and `min_Arab`, have no available training data, and hence we did not include them in the model training dataset.

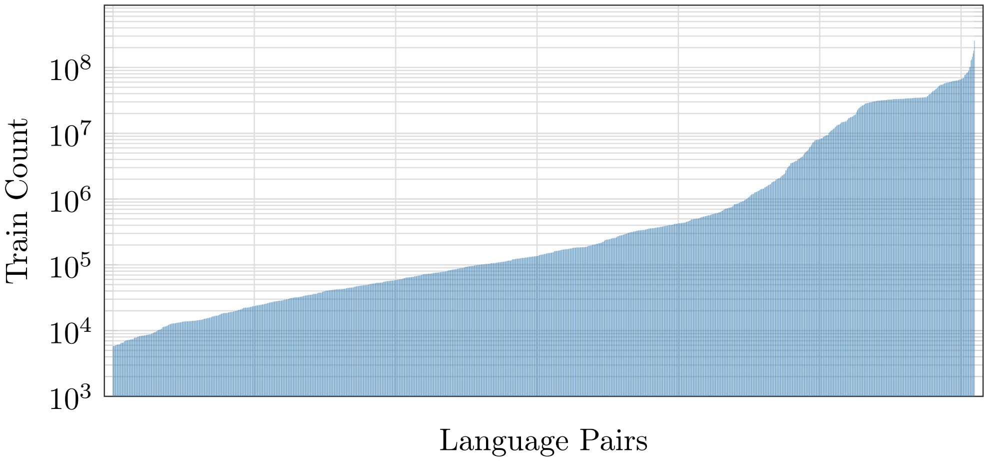


**Fig. 7**: Distribution of Amount of Training Sentence Pairs across 1220 language pairs in our dataset. We observe that the majority of pairs have fewer than 1M sentences and are low-resource.

pairs, and 261 directions of back-translated bitext. In total, there are **1220** language pairs or 2440 directions (`xx-yy` and `yy-xx`) for training. These 2440 directions result in over **18B** total sentence pairs. Figure 7 displays the distribution of samples across the 1220 language pairs—the majority of the pairs have fewer than 1M sentences and are low-resource directions.

# E Modeling

## E.1 Technical details

Both the encoder and decoder are stacks of Transformer layers. Each Transformer layer takes a sequence of embeddings as input and outputs a sequence of embeddings. In the encoder, Transformer layers are composed of two sub-layers, a self-attention and a feed-forward layer. These are applied sequentially and are both preceded by a LayerNorm [96] and followed by a residual connection [97]:

$$Z = X + \texttt{self-attention}(\texttt{norm}(X)), \tag{6}$$
$$Y = Z + \texttt{feed-forward}(\texttt{norm}(Z)). \tag{7}$$

We applied LayerNorm at the beginning of each sub-layer (Pre-LN) instead of applying LayerNorm after the residual connection at the end of each sub-layer (Post-LN). This is because Pre-LN is more stable in practice compared to Post-LN [98]. The self-attention layer is an attention layer that updates each element of the sequence by looking at the other elements, while the feed-forward layer (FFN) passes each element of the sequence

| Corpus Name | Citation | # Directions | # Languages |
|---|---|---|---|
| AAU Ethiopian Languages | Abate *et al.* [79] | 3 | 4 |
| AI4D | Degila *et al.* [80] and Siminyu *et al.* [81] | 3 | 5 |
| DGT | Tiedemann [54] | 94 | 24 |
| ECB | Tiedemann [54] | 74 | 19 |
| EMEA | Tiedemann [54] | 86 | 22 |
| English-Twi | Azunre *et al.* [82, 83] | 2 | 1 |
| EU Bookshop | Skadiņš *et al.* [84] | 160 | 38 |
| GlobalVoices | Tiedemann [54] | 235 | 41 |
| HornMT | Hadgu *et al.* [85] | 10 | . 5 |
| InfoPankki v1 | Tiedemann [54] | 30 | 12 |
| QCRI Educational Domain | Abdelali *et al.* [86] | 866 | 135 |
| JHU Bible | McCarthy *et al.* [23] | 300 | 155 |
| MADAR | Bouamor *et al.* [87] | 5 | 6 |
| Mburisano | Marais *et al.* [88] | 7 | 8 |
| MENYO-20k | Adelani *et al.* [89] | 2 | 1 |
| MultiIndicMT | Nakazawa *et al.* [90] | 10 | 11 |
| NLLB-Seed | *This work* | 39 | 40 |
| OpenSubtitles v2018 | Lison & Tiedemann [91] | 370 | 53 |
| Tanzil | Tiedemann [54] | 273 | 38 |
| Tatoeba | Tiedemann [54] | 493 | 143 |
| Tico19 v20201028 | Anastasopoulos *et al.* [92] | 48 | 34 |
| TWB-Gamayun | Oktem *et al.* [93] | 4 | 6 |
| United Nations Resolutions | Rafalovitch & Dale [94] | 20 | 7 |
| Turkic Interlingua (TIL) | Mirzakhalov *et al.* [95] | 46 | 11 |
| Wikimedia v20210402 | Tiedemann [54] | 582 | 154 |
| XhosaNavy | Tiedemann [54] | 2 | 1 |

**Table 5**: Summary of some of the main datasets used in training NLLB-200. Direction counts do not include reverse directions.

independently through a 2-layer MLP. In the decoder, there is an additional third sublayer between the self-attention and the feed-forward, which computes attention over the encoder output. We refer the reader to [63] for further details.

### *Sparsely gated mixture of experts*

As illustrated in Figure 8, we replaced the FFN sublayer in dense models with an MoE sublayer once every $f_{\text{MoE}}$ layers in both the encoder and decoder. The MoE sublayer consists of $E$ feed-forward networks (FFN), denoted with $(\text{FFN}_1, \text{FFN}_2, \ldots, \text{FFN}_E)$, each with input and output projections $W_i^{(e)}$ and $W_o^{(e)}$. A gating network, consisting of a softmax-normalized linear layer with weights $W_g$, is attached to each MoE sublayer to decide how to route tokens to experts. Given an input token $x_t$ the output of the MoE sublayer is evaluated as:

$$\text{FFN}_e(x_t) = W_o^{(e)}\,\text{ReLU}(W_i^{(e)} \cdot x_t), \quad (\forall e \in \{1, \ldots, E\}) \tag{8}$$

$$G_t = \text{softmax}(W_g \cdot x_t), \quad \mathcal{G}_t = \text{Top-k-Gating}(G_t), \tag{9}$$

$$\text{MoE}(x_t) = \sum_{e=1}^{E} \mathcal{G}_{te} \cdot \text{FFN}_e(x_t), \tag{10}$$

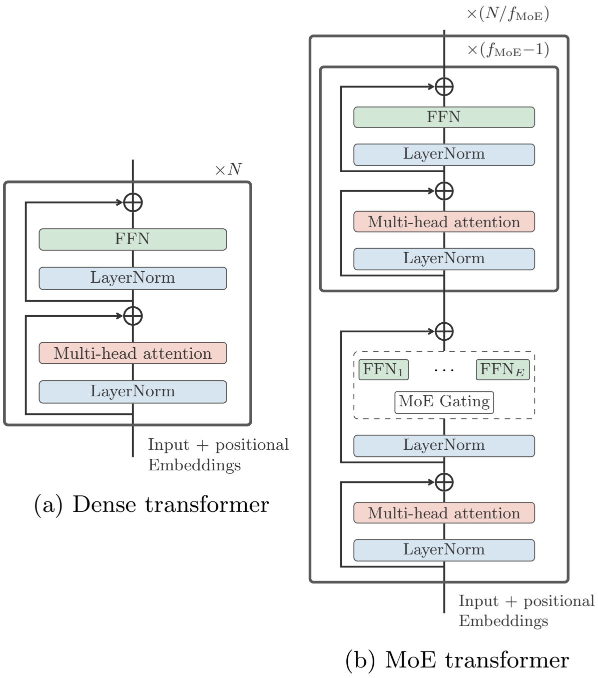


**Fig. 8**: Illustration of a Transformer encoder with MoE layers inserted at a 1:$f_{\text{MoE}}$ frequency. Each MoE layer has $E$ experts and a gating network responsible for dispatching tokens.

with $\mathcal{G}_t \in \mathbb{R}^E$ the routing vector computed by the gating network, i.e., for each expert, $\mathcal{G}_{t,e}$ is the contribution of the e$^{\text{th}}$ expert (FFN$_e$) in the MoE output. We followed the Top-k-Gating algorithm of [18] and dispatched each token to at most $k = 2$ experts. We always chose the top two scoring experts per token and did not add randomization to the choice of the second expert.

The Transformer encoder-decoder model, supplemented with MoE layers and their respective gating networks, learns to route input tokens to the corresponding top-two experts by optimizing a linearly weighted combination of label-smoothed cross entropy [39] and an auxiliary load balancing loss [20]. This additional loss term (LB) pushes the tokens to be uniformly distributed across experts and is evaluated as:

$$LB = E \cdot \sum_{e=1}^{E} f_e p_e, \quad p_e = \frac{1}{T} \cdot \sum_{t=1}^{T} G_{te}, \tag{11}$$

where $f_e$ is the fraction of tokens routed to the e$^{\text{th}}$ expert, as their first choice, through Top-k-Gating, and $p_e$ is the average routing probability to that expert over the $T$ tokens in the mini-batch. We refer the reader to [18] for more on the optimization of MoE models.

## E.2 Curriculum learning buckets

For the different curriculum setups, here is the list of directions used:

1. Step $0 - 170k$:
   https://github.com/facebookresearch/fairseq/tree/nllb/examples/nllb/modeling/scripts/flores200/final_lang_pairs_cl3.txt
2. Step $170k - 230k$:
   https://github.com/facebookresearch/fairseq/tree/nllb/examples/nllb/modeling/scripts/flores200/final_lang_pairs_cl2.txt
3. Step $230k - 270k$:
   https://github.com/facebookresearch/fairseq/tree/nllb/examples/nllb/modeling/scripts/flores200/final_lang_pairs_cl1.txt
4. Step $270k - 300k$:
   https://github.com/facebookresearch/fairseq/tree/nllb/examples/nllb/modeling/scripts/flores200/lang_pairs.txt

## E.3 Finetuning NLLB-200

Our goal in the next set of experiments is to examine if we are developing a robust general-purpose MT system capable of translating in various domains. For this purpose, we study if NLLB-200 can effectively transfer to other domains and if it lends itself to the common strategy of single-task finetuning with small quantities of in-domain high quality translations [99–102].

***Experimental Setup.***

We experimented with the NLLB-MD dataset (see Appendix J). It provides high-quality translations in four domains—news, scripted formal speech (scripted), unscripted informal speech (chat), and health. Language wise, it includes translations from English to six languages (five of which are low-resource). We held 500 sentences in each language for testing, finetuned on 2000 sentences, and used the remainder for validation. In each translation direction (into and out of English), we finetuned NLLB-200 on that single task for 50 updates (15-20 epochs) with a learning rate of 5e-5 following an inverse square-root schedule after warming up for ten updates. We considered two options for finetuning NLLB-200 for the new task: **(1)** finetuning with the original training objective (label-smoothed cross-entropy with an additional load balancing regularization term) and **(2)** finetuning without regularization and, thus, leaving the MoE's load distribution unconstrained.

***Results.***

Figure 9 shows validation chrF++ scores in the chat domain tasks of the pre-trained NLLB-200, the similarly finetuned model with load balancing (NLLB-200+FN+LB), and the finetuned model without load balancing (NLLB-200+FN).

On average, finetuning (FN+LB) improves the accuracy by +6.1 chrF++ points. The performance gain is more considerable when translating into high-resource languages (`eng` and `rus`), with an average +8.9 chrF++ points. When translating into the

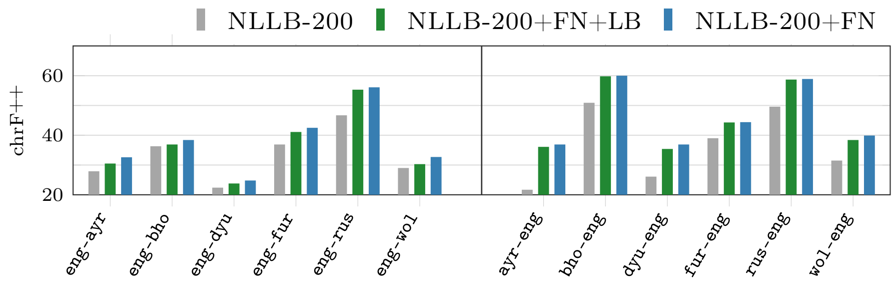


**Fig. 9**: Comparison of NLLB-200 with and without Finetuning on the 12 English-centric tasks of NLLB-MD. NLLB-200+FN+LB and +FN refer to finetuning with and without load balancing (LB). We report accuracy in terms of chrF++ on the validation set.

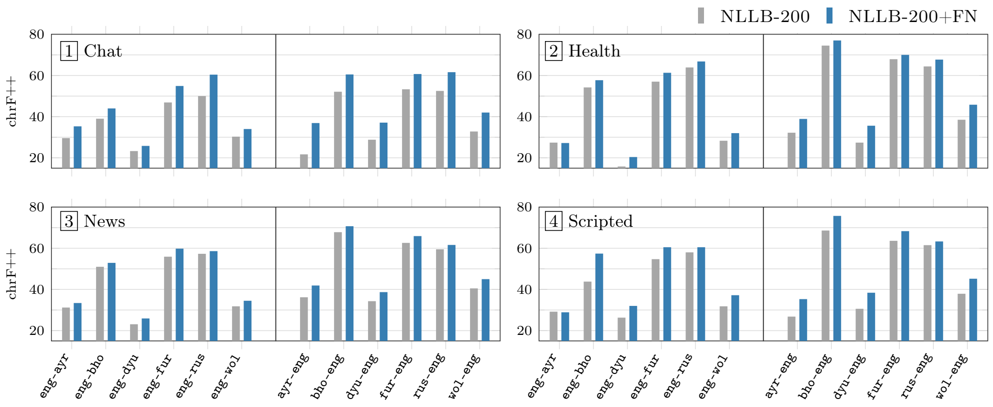


**Fig. 10**: Performance on NLLB-MD Test Sets (12 tasks in 4 domains) of NLLB-200 and the single-task finetuned models NLLB-200+FN (without load balancing).

five low-resource languages in NLLB-MD, the gain is 2.0 chrF++ points. When switching off the load balancing regularization, NLLB-200+FN improves by +7.2 chrF++ points. Particularly noteworthy is when translating into low-resource languages, which produces an increase of 3.7 points.

We next finetuned with our best strategy (NLLB-200+FN) on the other three domains of NLLB-MD and report chrF++ scores on the test sets in Figure 10. On average, by finetuning NLLB-200, we improved translation accuracy in the new domains by +7.7 in chat, +3.1 in news, +4.1 in health, and +5.8 in scripted (all in terms of chrF++). These results are evidence of NLLB-200's transferability and adaptability to other domains.

The issue of finetuning sparsely activated large models has been raised in prior work [21, 103, 104]. These large models are more prone to overfitting than their dense counterparts and, in some cases, perform poorly when finetuned [103, 104]. Fedus *et al.* [104] suggests increasing regularization with *expert dropout*, effectively applying

stronger regularization to the expert parameters, while Zoph *et al.* [21] combat overfitting by updating only a subset of model parameters. With MoE Expert Output Masking (EOM), NLLB-200 is heavily regularized and exhibits less overfitting on downstream tasks. We hypothesize that without load balancing, we allow the model to drop experts, practically activating a few that will be finetuned for the downstream task. This is particularly relevant when finetuning on a single task for which NLLB-200 has learned to assign specific experts (see section 8.5 from [34]); adding load balancing loss when the mini-batches are not mixed will considerably shift this learned assignment. We leave the exploration of MoE finetuning strategies with added regularization, selective fine-tuning, and relaxed optimization for future work.

# F Human evaluation details

***Annotators***

All evaluators were professional translators. Beyond this qualification, the standard requirements used were: 3+ years' translation experience in a language pair; native speaker fluency in the target language; high level in English (C2-C1).

***XSTS***

We adapted the recently proposed XSTS methodology from Agirre *et al.* [49]. In short, XSTS is a human evaluation protocol focusing on *meaning preservation* above fluency. For low-resource languages, translations are usually of poorer quality, and so we focus on usable (i.e., meaning-preserving) translations, even if they are not fully fluent. Compared to Direct Assessment [72] with a 5-point scale (the original direct assessment uses a 100-point scale), work has found that XSTS yields higher inter-annotator agreement [48].

XSTS rates each source sentence and its machine translation on a five-point scale, where one is the lowest and five is the highest. Each point on the scale is as follows:

1. The two sentences are not equivalent, share few details and may be about different topics. If the two sentences are about similar topics, but less than half of the core concepts mentioned are the same, 1 is still the appropriate score.
2. The two sentences share some details but are not equivalent. Some important information related to the primary subject/verb/object differs or is missing, which alters the intent or meaning of the sentence.
3. The two sentences are mostly equivalent, but some unimportant details can differ. There cannot be any significant conflicts in intent or meaning between the sentences, no matter how long the sentences are.
4. The two sentences are paraphrases of each other. Their meanings are near-equivalent, with no major differences or missing information. There can only be minor differences in meaning due to differences in expression (e.g., formality level, style, emphasis, potential implication, idioms, common metaphors).
5. The two sentences are precisely and completely equivalent in meaning and usage expression (e.g., formality level, style, emphasis, potential implication, idioms, common metaphors).

Further details on calibration are reported in section 7.2 of [34].

# G Limitations

In the previous sections, we documented how several data, modeling, and evaluation challenges were overcome to realize NLLB-200. In this section, we underline some limitations in our effort.

### *Bitext mining for low-resource languages*

For some languages, we could only create a small amount of bitext through data mining. The main limiting factor lies in the paucity of monolingual data. More specifically, many low-resource languages have a limited web presence, and even though the data we curated was processed across many stages (i.e., language identification, aggressive cleaning of monolingual data, etc.), the amount of training data for different languages remained unbalanced. An important final consideration is the web is saturated with machine-translated content. For example, many websites may use translation to localize their content. On the upside, most of the languages we targeted in NLLB-200 are not supported by most existing commercial translation services. However, in the process of mining higher-resource languages, it is likely that our mined data sets contain pre-translated content.

We also want to reflect on the issue of data ownership. In an interview study we conducted with low-resource language speakers, many participants expressed that sharing language access might, in fact, be a necessary trade-off for technological advancement. Blocking such access meant blocking any future benefits that could positively impact low-resource language communities. However, we stress that access and ownership are two disparate concepts. Even though we deploy many low-resource language data sets, ownership ultimately belongs to the speakers of these languages.

### *Pairing self-supervised learning with machine translation*

Recent work [75, 76, 105] demonstrates that denoising and similar self-supervised objectives are very useful for improving model performance when trained concomitantly with machine translation tasks in a multitask setup. In NLLB-200, we tried two self-supervised learning (SSL) objectives and experimented with different combinations of both alongside the MMT task. We observe that only denoising autoencoder (DAE) performs well when trained with MMT. The benefits of the LM task in a multitask setup with MMT are not well-studied, and future work could reveal a deeper understanding of the mechanisms supporting this finding.

### *Deploying translation models for specific domains or language families*

Practically deploying machine learning models is technically challenging and remains an active area of research. Our investigation indicates that distillation is a promising avenue for leveraging multilingual models and adapting them to a subset of desired language directions and domains. This has allowed the Wikipedia translation model trained in NLLB Team et al. [34] to perform better than much larger models. In the same paper, we also demonstrated multidialectal translation capabilities by translating from and into different Arabic languoids. We found that while a massive multilingual model achieves the best average score, a smaller specialized model outperforms the

former in specific directions. This highlights the importance of more focused research on closely related languages.

### *Curating benchmark datasets for low-resource languages*

Compared to creating FLORES-101, our new translation workflow substantially streamlined the process of realizing FLORES-200. For example, the number of languages requiring re-translation in FLORES-200 was ten, down from 45 in its predecessor. However, despite these improvements, we continued to experience similar difficulties to those of FLORES-101, but at an even greater scale due to the increasingly low-resource nature of the supported languages. Moreover, industry-wide standards for dealing with these lower-resource languages are limited, leading to more logistical barriers for us to navigate [84]. This led to longer turnaround times, occasionally forced by the need to find new translators and reviewers. In the cases of Sicilian and Buginese, work on these languages took significantly longer than other languages to complete (287 days).

### *XSTS for human evaluation*

XSTS scoring followed by calibration successfully addresses the issues of evaluation consistency across evaluators and language directions in a massively multilingual context. However, as this metric is focused on meaning preservation rather than fluency, it may face difficulties when used to evaluate the quality of translations across coexisting language registers.

### *Added toxicity detection*

Detecting added toxicity remains challenging, especially when detection must be done at scale for 200 languages. Since we evaluated our approach on a translated data set, the quality of translations may be a confounding factor worth exploring. For example, the quality of the toxicity detection can be affected by the amount of resources available per language. Alternatively, the quality and efficiency of our detectors, which locate or filter toxicity, may vary depending on list-building inconsistencies, list length, segmentation accuracy, the degree of complexity in morphological variation, and the amount of non-lexicalized toxicity. The expansion and disambiguation of small toxicity lists are critical areas for future work, which likely require close collaboration with a larger number of native speakers. A first step towards disambiguation can be contextualizing polysemous words by replacing single tokens with n-grams that have a much higher probability of representing true toxic content. Finally, we know that added toxicity can be caused by phenomena that would be considered instances of hallucination. Our visualization examples with ALTI+, which show a low amount of source contribution in toxicity when computed with this method, are a strong indicator of hallucination. Additional work aiming to further quantify and mitigate added toxicity is already in progress [106].

# H Model Card - NLLB-200

## Model Details[a]

- Person or organization developing model: *Developed by Meta AI Research*
- Model date: *June 30th, 2022*
- Model version: NLLB-200
- Model type: *Transformer Mixture-of-Experts machine translation model.*
  - Information about training algorithms, parameters, fairness constraints or other applied approaches, and features *The exact training algorithm, data, and the strategies to handle data imbalances for high and low resource languages that were used to train* NLLB-200 *is described in the paper.* NLLB Team et al., *No Language Left Behind: Scaling Human-Centered Machine Translation*, arXiv, 2022
  - License: *CC-BY-NC*[b]
  - Where to send questions or comments about the model: https://github.com/facebookresearch/fairseq/issues

## Intended Use

- Primary intended uses: NLLB-200 *is a machine translation model primarily intended for research in machine translation, especially for low-resource languages. It allows for single-sentence translation among 200 languages. Information on how to use the model can be found in Fairseq code repository, along with the training code and references to evaluation and training data.*
- Primary intended users: *Primary users are researchers and the machine translation research community.*
- Out-of-scope use cases: NLLB-200 *is a research model and is not released for production deployment.* NLLB-200 *is trained on general domain text data and is not intended to be used with domain-specific texts, such as medical or legal domains. The model is not intended to be used for document translation. The model was trained with input lengths not exceeding 512 tokens. Therefore, translating longer sequences might result in quality degradation.* NLLB-200 *translations can not be used as certified translations.*

## Metrics

- Model performance measures: NLLB-200 *model was evaluated using BLEU, spBLEU, and chrF++ metrics widely adopted by machine translation community. Additionally, we performed human evaluations with the XSTS protocol and measured the toxicity of the generated translations.*

## Evaluation Data

- Datasets: FLORES-200 *dataset is described in section 4 of the paper.*
- Motivation: *We used* FLORES-200 *as it provides full evaluation coverage of the languages in* NLLB-200.
- Preprocessing: *Sentence-split raw text data was preprocessed using SentencePiece. The SentencePiece model is released along with* NLLB-200.

## Training Data

- *We used parallel multilingual data from various sources to train the model. We provide a detailed report on the data selection and construction process in section 2 of the paper. We also used monolingual data constructed from Common Crawl. We provide more details in section 5.2 of the paper*

## Ethical Considerations

- *In this work, we took a reflexive approach in technological development to ensure that we prioritize human users and minimize risks that could be transferred to them. While we reflect on our ethical considerations throughout the article, here are some additional points to highlight. For one, many languages chosen for this study are low-resource languages, with a heavy emphasis on African languages. While quality translation could improve education and information access in many of these communities, such access could also make groups with lower levels of digital literacy more vulnerable to misinformation or online scams. The latter scenarios could arise if bad actors misappropriate our work for nefarious activities, which we conceive as an example of unintended use. Regarding data acquisition, the training data used for model development were mined from various publicly available sources on the web. Although we invested heavily in data cleaning, personally identifiable information may not be entirely eliminated. Finally, although we did our best to optimize for translation quality, mistranslations produced by the model could remain. Although the odds are low, this could have an adverse impact on those who rely on these translations to make important decisions (particularly when related to health and safety).*

## Caveats and Recommendations

- *Our model has been tested on the Wikimedia domain with a limited investigation on other domains supported in* NLLB-MD. *In addition, the supported languages may have variations that our model is not capturing. Users should make appropriate assessments.*

## Carbon Footprint Details

- *The carbon dioxide ($CO_2e$) estimate is reported in section 8.8 of the paper.*

---

[a] For this card, we used the template from [107].
[b] https://creativecommons.org/licenses/by-nc/4.0/legalcode

# I Data Card for NLLB-Seed Data

**Dataset Description**[a]

- Dataset Summary
  *The* NLLB-Seed *data is a collection of human-translated data sampled from Wikimedia' s List of articles every Wikipedia should have*[b]*, a collection of 10,000 Wikidata IDs corresponding to notable topics in different fields of knowledge and human activity. It contains bitext from English to 43 languages in 6193 sentences. The motivation of this data was to provide a starter set of clean data on a variety of topics in those languages.*

- How to use the data
  You can access links to the data in the README at https://github.com/facebookresearch/fairseq/tree/nllb

- Supported Tasks and Leaderboards
  *NLLB model uses this data to boost the performance of low-resource languages.*

- Languages
  NLLB-Seed *contains 43 language pairs with English.*

**Dataset Creation**

- Curation Rationale
  *Script, dialect, spelling, and translation approaches were first established and aligned on from* Flores-200. *Translators referenced these linguistic alignments while working on* NLLB-Seed *translations. The data sets were translated directly from English for 39 languages; half the data for Ligurian (3000 sentences) were first translated from English to Italian, then translated from Italian to Ligurian while the other half was translated directly from English, and three Arabic script languages (Acehnese, Banjar, Tamasheq) were transliterated from their respective Latin script datasets that were translated from English. Following the translation or transliteration phase was a linguistic quality assessment phase in which the completed data sets were checked against the linguistic alignments from* Flores-200 *along with basic quality sanity checks. The data sets were then finalized and completed.*

- Source Data
  *Source Data includes 6193 English sentences sampled from Wikipedia Articles in 11 categories: Anthropology, Arts, Biology, Geography, History, Mathematics, People, Philosophy, Physical, Society, Technology.*

- Annotations
  *There are no extra annotations with the bitext.*

- Personal and Sensitive Information
  *Not applicable*

**Considerations for Using the Data**

- Social Impact of Dataset
  *The dataset is specifically built to increase the translation quality and improve language identification of the extremely low-resource languages it contains. This helps improve the quality of different languages in machine translation systems.*

- Discussion of Biases
  *Biases on the dataset have not been studied.*

**Additional Information**

- Dataset Curators
  *All translators who participated in the* NLLB-Seed *data creation underwent a vetting process by our translation vendor partners. Translators are required to be native speakers and educated in the target language. They must also have a high level of fluency (C1-C2) in English. For non-English translators, they are required to have a high level of fluency in their source language. Translators were also required to have at least two to three years of translation experience in the relevant language pair if they have an academic degree in translation or linguistics and three to five years of translation experience if they do not have any relevant academic qualification. Translators also undergo a translation test every 18 months to assess the quality of their abilities.*

- Licensing Information
  *We are releasing translations based on source sentences from Wikipedia under the terms of CC-BY-SA*[c]

- Citation Information
  NLLB Team et al., *No Language Left Behind: Scaling Human-Centered Machine Translation*, arXiv, 2022

[a] We use a template for this data card https://huggingface.co/docs/datasets/v1.12.0/dataset_card.html
[b] https://meta.wikimedia.org/wiki/List_of_articles_every_Wikipedia_should_have/Expanded
[c] https://creativecommons.org/licenses/by-sa/4.0/

# J Data Card for NLLB Multi-Domain Data

**Dataset Description**[a]

- Dataset Summary
  *The NLLB Multi-Domain data is a collection of human-translated data across four domains (11810 sentences across news, formal speech, informal speech, and medical sources). It contains bitext from English to other six languages. The motivation of this data was to help improve model performance on text from different domains and assess how well a general translation model can be fine-tuned on a dataset covering a new domain.*
- How to use the data
  You can access links to the data in the README at https://github.com/facebookresearch/fairseq/tree/nllb
- Supported Tasks and Leaderboards
  *NLLB model uses this data to boost the performance of low-resource languages.*
- Languages
  *NLLB Multi-Domain contains 6 language pairs with English: Central Aymara (`ayr_Latn`), Bhojpuri (`bho_Deva`), Dyula (`dyu_Latn`), Friulian (`fur_Latn`), Russian (`rus_Cyrl`) and Wolof (`wol_Latn`).*

**Data Structure**

- *The data set contains gzipped tab delimited text files for each direction. Each text file contains lines with parallel sentences. The data is not split.*

**Data Set Creation**

- Curation Rationale
  *Script, dialect, spelling, and translation approaches were first established and aligned on from* Flores-200*. Translators referenced these linguistic alignments while working on NLLB Multi-Domain data translations. The data sets were translated directly from English for all six languages, followed by a linguistic quality assessment phase in which the completed datasets were checked against the linguistic alignments from* Flores-200 *along with basic quality sanity checks. The data sets were then finalized and completed.*
- Source Data
  *Source Data includes three domains:*
  - *News: 2810 English sentences from the WMT21 English-German development set, containing a sample of newspapers from 2020 [46]*
  - *Unscripted Informal Speech: 3000 English utterances from the multi-session chat dataset of Xu* et al. *[108], which contains on average 23 words per turn*
  - *Health: 3000 English sentences from a World Health Organization report [109] and the English portion of the TAUS Corona Crisis Report.*[b]*)*
- Annotations
  *There are no extra annotations with the bitext.*
- Personal and Sensitive Information
  *Not applicable*

**Considerations for Using the Data**

- Social Impact of Data Set
  *The data set is specifically built to increase the translation quality and the language identification of the extremely low-resource languages it contains. This helps improve the quality of different languages in machine translation systems.*
- Discussion of Biases(#discussion-of-biases)
  *Biases on the data set have not been studied.*

**Additional Information**

- Dataset Curators
  *All translators who participated in the NLLB Multi-Domain data creation underwent a vetting process by our translation vendor partners. Translators are required to be native speakers and educated in the target language. They must also have a high level of fluency (C1-C2) in English. For non-English translators, they are required to have a high level of fluency in their source language. Translators must also have at least two to three years of translation experience in the relevant language pair if they have an academic degree in translation or linguistics and three to five years of translation experience if they do not have any relevant academic qualification. Translators undergo a translation test every 18 months to assess their translation capabilities and have it for reference for all future projects.*
- Licensing Information
  *We are releasing translations based on source sentences from the World Health Organization under the terms of CC-BY-SA.*[c] *We are releasing translations based on source sentences from TAUS, Multi-Session Chat, and WMT under the terms of CC-BY-NC.*[d]
- Citation Information
  NLLB Team et al., *No Language Left Behind: Scaling Human-Centered Machine Translation*, arXiv, 2022

---

[a] We use a template for this data card https://huggingface.co/docs/datasets/v1.12.0/dataset_card.html. Note that this card overlaps significantly with the previous NLLB-Seed card.
[b] https://md.taus.net/corona
[c] https://creativecommons.org/licenses/by-sa/4.0/
[d] https://creativecommons.org/licenses/by-nc/4.0/legalcode

# K Data Card for Mined Bitext Metadata

**Dataset Description**[a]

- Dataset Summary
  *We created mined bitext from publicly available web data for 148 English-centric and 1465 non-English-centric language pairs using the stopes mining library and the LASER3 encoders* Heffernan et al. *[56]. We open-source the corresponding metadata and a script that enables researchers who have downloaded the specified files from CommonCrawl and ParaCrawl to recreate the full bitext data. Note that CommonCrawl answers takedown notices, so subsequent runs of the tool can end up with a smaller amount of bitext.*
- How to use the data
  You can access links to the data in the README at https://github.com/facebookresearch/fairseq/tree/nllb

**Data Structure**

- *The metadata files are space-separated, xz-compressed files. Each file corresponds to one bitext direction. For example, the file* `xho_Latn-yor_Latn.meta.xz` *contains all the metadata required to find the actual Xhosa and Yoruba-aligned text data. Each line has 11 columns with the following format:*
  - *If the metadata comes from Common Crawl:* `wet_file_url document_sha1 document_url line_number_in_document paragraph_digest sentence_digest lid_score laser_score direction language line_number_in_direction`
  - *If the metadata comes from other corpus:* `corpus_name.language not_used not_used line_number_in_document paragraph_digest sentence_digest lid_score laser_score direction language line_number_in_direction`
- *Paragraph and sentence digests are computed with* `xxh3_64_intdigest`.

**Data Splits**

- *Given the noisy nature of the overall process, we recommend using the data only for training and use other datasets like* Flores-200 *for the evaluation.*

**Dataset Creation**

- Source Data
  *Initial Data Collection and Normalization The monolingual data is from Common Crawl and ParaCrawl.*
- Curation Rationale
  *We applied filtering based on language identification, emoji-based filtering, and language model-based filtering for some high-resource languages. For more details on our data filtering, please refer to section 5.2 of the paper.*
- Who are the source language producers?
  *The source language was produced by writers of each website that Common Crawl and ParaCrawl have crawled.*
- Annotations
  - Annotation process
    *Parallel sentences in the monolingual data were identified using LASER3 encoders. [56]*
  - Who are the annotators?
    *The data was not human annotated.*
  - Personal and Sensitive Information
    *The metadata files do not contain any text beyond website URLs. However, the data in CommonCrawl and ParaCrawl may contain personally identifiable information, or sensitive or toxic content that was publicly shared on the Internet. Some of this information may have been referred to in the released data set.*

**Considerations for Using the Data**

- Social Impact of Dataset
  *This data can be used to reconstruct a dataset for training machine learning systems for many low-resource languages.*
- Discussion of Biases
  *Biases in the data have not been specifically studied. However, as the original data source is the World Wide Web, it is likely that the data has biases similar to those prevalent in the Internet. The data may also exhibit biases introduced by language identification and data filtering techniques. As such, lower-resource languages may have lower accuracy.*

**Additional Information**

- Data set Curators
  *The data was not curated*
- Licensing Information
  *We are releasing the metadata and the script to recreate the bitext from it under the terms of CC-BY-NC.*[b] *The text and copyright (where applicable) remain with the original authors or publishers, please adhere to the applicable licenses provided by the original authors. We keep track of the source URL of each individual sentence to allow people to refer to the said website for licensing information.*
- Citation Information
  NLLB Team et al., *No Language Left Behind: Scaling Human-Centered Machine Translation*, arXiv, 2022

[a] For this card we use the template available https://huggingface.co/docs/datasets/v1.12.0/dataset_card.html. We provide details on the metadata released.
[b] https://creativecommons.org/licenses/by-nc/4.0/legalcode

## References


73. Gu, J., Wang, Y., Cho, K. & Li, V. O. *Improved Zero-shot Neural Machine Translation via Ignoring Spurious Correlations* in *Proceedings of the 57th Annual Meeting of the Association for Computational Linguistics* (2019), 1258–1268.
74. Chen, G. *et al.* Towards Making the Most of Multilingual Pretraining for Zero-Shot Neural Machine Translation. *CoRR* **abs/2110.08547.** arXiv: 2110.08547. https://arxiv.org/abs/2110.08547 (2021).
75. Bapna, A. *et al. Building Machine Translation Systems for the Next Thousand Languages* 2022. https://arxiv.org/abs/2205.03983.
76. Ma, S. *et al.* DeltaLM: Encoder-Decoder Pre-training for Language Generation and Translation by Augmenting Pretrained Multilingual Encoders. *CoRR* **abs/2106.13736.** arXiv: 2106.13736. https://arxiv.org/abs/2106.13736 (2021).
77. Liu, Y. *et al.* Multilingual denoising pre-training for neural machine translation. *Transactions of the Association for Computational Linguistics* **8,** 726–742 (2020).
78. Volansky, V., Ordan, N. & Wintner, S. On the features of translationese. *Digital Scholarship in the Humanities* **30,** 98–118 (2015).
79. Abate, S. T. *et al. Parallel Corpora for bi-Directional Statistical Machine Translation for Seven Ethiopian Language Pairs* in *Proceedings of the First Workshop on Linguistic Resources for Natural Language Processing* (2018), 83–90.
80. Degila, K., Kalipe, G., Ali, J. T. & Balogoun, M. *Parallel text dataset for Neural Machine Translation (French -> Fongbe, French -> Ewe)* version 1.0. Nov. 2020. https://doi.org/10.5281/zenodo.4266935.
81. Siminyu, K. *et al.* AI4D – African Language Program. *arXiv preprint arXiv:2104.02516* (2021).
82. Azunre, P. *et al.* English-Twi Parallel Corpus for Machine Translation. *arXiv preprint arXiv:2103.15625* (2021).
83. Azunre, P. *et al. ENGLISH-AKUAPEM TWI PARALLEL CORPUS* version 1.0.1. Jan. 2021. https://doi.org/10.5281/zenodo.4432117.
84. Skadiņš, R., Tiedemann, J., Rozis, R. & Deksne, D. *Billions of Parallel Words for Free: Building and Using the EU Bookshop Corpus* in *Proceedings of the Ninth International Conference on Language Resources and Evaluation (LREC'14)* (European Language Resources Association (ELRA), Reykjavik, Iceland, May 2014), 1850–1855.
85. Hadgu, A. T., Gebremeskel, G. G. & Aregawi, A. *HornMT: Machine Translation Benchmark Dataset for Languages in the Horn of Africa* https://github.com/asmelashteka/HornMT. 2021.
86. Abdelali, A., Guzman, F., Sajjad, H. & Vogel, S. *The AMARA Corpus: Building Parallel Language Resources for the Educational Domain* in *Proceedings of the Ninth International Conference on Language Resources and Evaluation (LREC'14)* (European Language Resources Association (ELRA), Reykjavik, Iceland, May 2014), 1856–1862. http://www.lrec-conf.org/proceedings/lrec2014/pdf/877_Paper.pdf.

87. Bouamor, H., Hassan, S. & Habash, N. *The MADAR Shared Task on Arabic Fine-Grained Dialect Identification* in *Proceedings of the Fourth Arabic Natural Language Processing Workshop* (Association for Computational Linguistics, Florence, Italy, Aug. 2019), 199–207. https://aclanthology.org/W19-4622.
88. Marais, L., Wilken, I., Van Niekerk, N. & Calteaux, K. *Mburisano Covid-19 multilingual corpus* https://hdl.handle.net/20.500.12185/536. 2021.
89. Adelani, D. *et al. The Effect of Domain and Diacritics in Yoruba–English Neural Machine Translation* in *Proceedings of the 18th Biennial Machine Translation Summit (Volume 1: Research Track)* (Association for Machine Translation in the Americas, Virtual, Aug. 2021), 61–75. https://aclanthology.org/2021.mtsummit-research.6.
90. Nakazawa, T. *et al. Overview of the 8th Workshop on Asian Translation* in *Proceedings of the 8th Workshop on Asian Translation (WAT2021)* (Association for Computational Linguistics, Online, Aug. 2021), 1–45. https://aclanthology.org/2021.wat-1.1.
91. Lison, P. & Tiedemann, J. *Opensubtitles2016: Extracting large parallel corpora from movie and tv subtitles* 2016.
92. Anastasopoulos, A. *et al. TICO-19: the Translation Initiative for COvid-19* in *Proceedings of the 1st Workshop on NLP for COVID-19 (Part 2) at EMNLP 2020* (Association for Computational Linguistics, Online, Dec. 2020). https://aclanthology.org/2020.nlpcovid19-2.5.
93. Oktem, A., Jaam, M. A., DeLuca, E. & Tang, G. *Gamayun – Language Technology for Humanitarian Response* in *2020 IEEE Global Humanitarian Technology Conference (GHTC)* (2020), 1–4.
94. Rafalovitch, A. & Dale, R. *United Nations General Assembly Resolutions: A Six-Language Parallel Corpus* in *Proceedings of the MT Summit XII* (Ottawa, Canada, 2014), 292–299.
95. Mirzakhalov, J. *et al.* A Large-Scale Study of Machine Translation in the Turkic Languages. *arXiv preprint arXiv:2109.04593* (2021).
96. Ba, L. J., Kiros, J. R. & Hinton, G. E. Layer Normalization. *CoRR* **abs/1607.06450.** arXiv: 1607.06450. http://arxiv.org/abs/1607.06450 (2016).
97. He, K., Zhang, X., Ren, S. & Sun, J. *Deep Residual Learning for Image Recognition* in *Proc. of CVPR* (2015).
98. Xiong, R. *et al. On layer normalization in the transformer architecture* in *International Conference on Machine Learning* (2020), 10524–10533.
99. Tang, Y. *et al.* Multilingual Translation with Extensible Multilingual Pretraining and Finetuning. *CoRR* **abs/2008.00401.** arXiv: 2008.00401. https://arxiv.org/abs/2008.00401 (2020).
100. Liu, Z., Winata, G. I. & Fung, P. *Continual Mixed-Language Pre-Training for Extremely Low-Resource Neural Machine Translation* in *Findings of the Association for Computational Linguistics: ACL-IJCNLP 2021* (Association for Computational Linguistics, Online, Aug. 2021), 2706–2718. https://aclanthology.org/2021.findings-acl.239.
101. Lee, E.-S. *et al. Pre-Trained Multilingual Sequence-to-Sequence Models: A Hope for Low-Resource Language Translation?* in *Findings of the Association for*

*Computational Linguistics: ACL 2022* (Association for Computational Linguistics, Dublin, Ireland, May 2022), 58–67. https://aclanthology.org/2022.findings-acl.6.

102. Adelani, D. I. *et al.* A Few Thousand Translations Go a Long Way! Leveraging Pre-trained Models for African News Translation. *CoRR* **abs/2205.02022.** arXiv: 2205.02022. https://arxiv.org/abs/2205.02022 (2022).
103. Artetxe, M. *et al.* Efficient Large Scale Language Modeling with Mixtures of Experts. *CoRR* **abs/2112.10684.** arXiv: 2112.10684. https://arxiv.org/abs/2112.10684 (2021).
104. Fedus, W., Zoph, B. & Shazeer, N. Switch Transformers: Scaling to Trillion Parameter Models with Simple and Efficient Sparsity. *Journal of Machine Learning Research* **23,** 1–39. http://jmlr.org/papers/v23/21-0998.html (2022).
105. Chi, Z. *et al.* mT6: Multilingual pretrained text-to-text transformer with translation pairs. *arXiv preprint arXiv:2104.08692* (2021).
106. Costa-jussà, M. R. *et al.* Toxicity in Multilingual Machine Translation at Scale. *arXiv preprint arXiv:2210.03070* (2022).
107. Mitchell, M. *et al.* *Model Cards for Model Reporting* in *Proceedings of the Conference on Fairness, Accountability, and Transparency* (Association for Computing Machinery, 2019), 220–229. ISBN: 9781450361255. https://doi.org/10.1145/3287560.3287596.
108. Xu, J., Szlam, A. & Weston, J. *Beyond Goldfish Memory: Long-Term Open-Domain Conversation* in *Proceedings of the 60th Annual Meeting of the Association for Computational Linguistics (Volume 1: Long Papers)* (Association for Computational Linguistics, Dublin, Ireland, May 2022), 5180–5197. https://aclanthology.org/2022.acl-long.356.
109. Donaldson, L. & Rutter, P. *Healthier, fairer, safe: the global health journey 2007–2017* tech. rep. (World Health Organization, May 2017).

## List of Supplementary Tables

4 No Language Left Behind languages: We display the language *Code*, language name, *Script*, and language *Family*. The symbol 🌐 indicates machine translation support by Google and/or Microsoft (as of July 2022), whereas ✗ indicates support by neither. *Res.* indicates if we classify the language as high or low-resource. *Specification* contains, if available, additional information on the language variant collected in FLORES-200. The superscript NEW indicates new languages added to FLORES-200 compared to FLORES-101. . . . . . . 33
5 Summary of some of the main datasets used in training NLLB-200. Direction counts do not include reverse directions. . . . . . . . . . . 39

## List of Supplementary Figures

6 Comparing model performance when trained on data from various sources. We observe significant improvements in adding mined and back-translated data for all types of language pairs and resource levels. 37

7 Distribution of Amount of Training Sentence Pairs across 1220 language pairs in our dataset. We observe that the majority of pairs have fewer than 1M sentences and are low-resource. . . . . . . . . . . . . . . . . . . 38
8 Illustration of a Transformer encoder with MoE layers inserted at a 1:$f_{\text{MoE}}$ frequency. Each MoE layer has $E$ experts and a gating network responsible for dispatching tokens. . . . . . . . . . . . . . . . . . . . . . 40
9 Comparison of NLLB-200 with and without Finetuning on the 12 English-centric tasks of NLLB-MD. NLLB-200+FN+LB and +FN refer to finetuning with and without load balancing (LB). We report accuracy in terms of chrF++ on the validation set. . . . . . . . . . . . . . . . . . . . . . . . . 42
10 Performance on NLLB-MD Test Sets (12 tasks in 4 domains) of NLLB-200 and the single-task finetuned models NLLB-200+FN (without load balancing). 42
